# Multimodal Brain Network Jointly Construction and Fusion for Diagnosis of Epilepsy

**DOI:** 10.3389/fnins.2021.734711

**Published:** 2021-09-29

**Authors:** Qi Zhu, Jing Yang, Bingliang Xu, Zhenghua Hou, Liang Sun, Daoqiang Zhang

**Affiliations:** ^1^College of Computer Science and Technology, Nanjing University of Aeronautics and Astronautics, Nanjing, China; ^2^Department of Psychosomatics and Psychiatry, Affiliated Zhongda Hospital, School of Medicine, Southeast University, Nanjing, China

**Keywords:** brain network analysis, node importance, multi-modal brain network, PageRank algorithm, disease diagnosis

## Abstract

Brain network analysis has been proved to be one of the most effective methods in brain disease diagnosis. In order to construct discriminative brain networks and improve the performance of disease diagnosis, many machine learning–based methods have been proposed. Recent studies show that combining functional and structural brain networks is more effective than using only single modality data. However, in the most of existing multi-modal brain network analysis methods, it is a common strategy that constructs functional and structural network separately, which is difficult to embed complementary information of different modalities of brain network. To address this issue, we propose a unified brain network construction algorithm, which jointly learns both functional and structural data and effectively face the connectivity and node features for improving classification. First, we conduct space alignment and brain network construction under a unified framework, and then build the correlation model among all brain regions with functional data by low-rank representation so that the global brain region correlation can be captured. Simultaneously, the local manifold with structural data is embedded into this model to preserve the local structural information. Second, the PageRank algorithm is adaptively used to evaluate the significance of different brain regions, in which the interaction of multiple brain regions is considered. Finally, a multi-kernel strategy is utilized to solve the data heterogeneity problem and merge the connectivity as well as node information for classification. We apply the proposed method to the diagnosis of epilepsy, and the experimental results show that our method can achieve a promising performance.

## 1. Introduction

Brain network analysis has been widely applied to analysis and diagnosis of brain diseases, such as epilepsy and Alzheimer's disease (Osipowicz et al., 2016). It mainly benefits from more and more neuroimaging technologies that can give us insight into the neuroanatomical correlates of cognition. functional MRI (fMRI) and diffusion tensor imaging (DTI) are of remarkable importance and widely used to construct brain networks (Osipowicz et al., 2016). Inspired by graph theory, brain network abstracted as a set of nodes and edges, is developed to describe the correlation or interaction among the different regions of the brain. In brain network, nodes represent region-of-interest (ROIs), and the edges between nodes represent the correlation between different brain regions (Fornito et al., 2016). Functional MRI (fMRI) can reflect temporal correlations between BOLD signals in brain regions, while diffusion tensor imaging (DTI) can be used to reveal the physical connectivity between the functionally relevant gray matter regions (Osipowicz et al., 2016).

According to Fornito et al. (2016), even a slight disruption in the small-world character of the functional brain networks would suggest a disruption in the integrity of the cognitive state systems involved in causing the disease. In practical application, functional connectivity (FC) can be constructed from fMRI and structural connectivity (SC) can be constructed from DTI. So that the FC can be used to detect the consistency of brain activities while the SC can measure neural fiber physical connections between different brain regions (Huang et al., 2019). It is widely acknowledged that both FC and SC are able to contribute significant information for brain disease diagnosis. Many recent researches have proved that combining the two modalities to construct brain network is an promising technique. Specifically, compared to the single-modal brain network, the multi-modal brain network can achieve better analysis and diagnosis results (Song et al., 2020).

**Figure 1:**
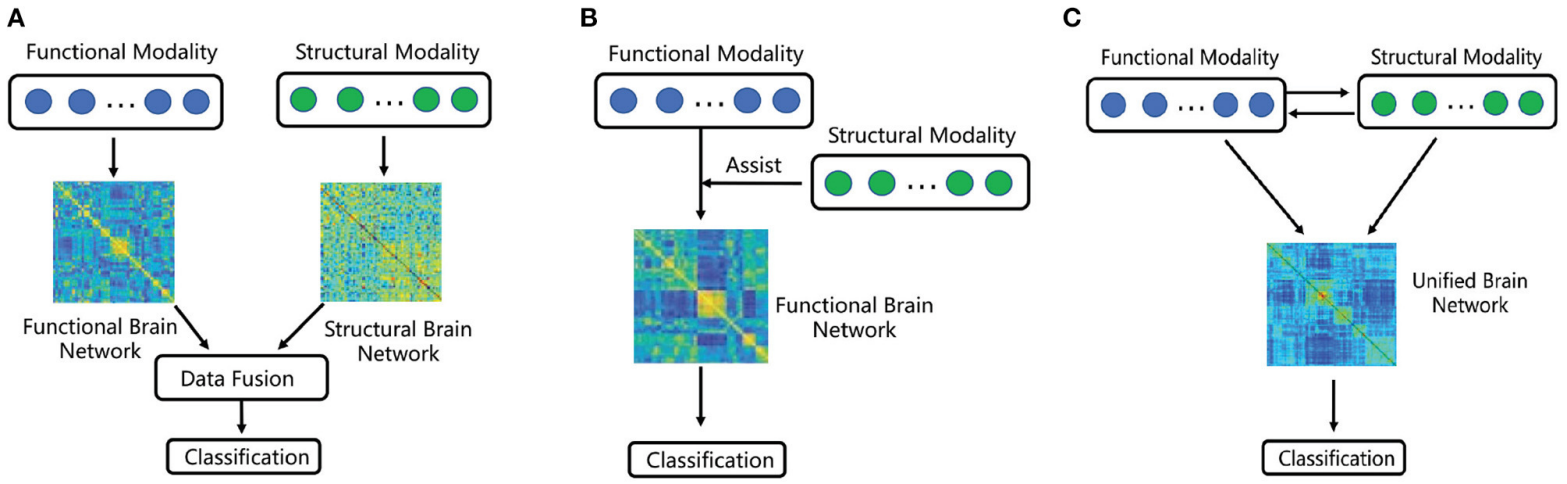
A sketched comparison between existing fusion methods and ours. **(A)** Multi-modal brain network based on feature integration. **(B)** Multi-modal brain network based on structural feature embedding. **(C)** Proposed multi-modal brain network fusion based on information interaction.

But majority of existing network-based analysis, which fuse FC and SC, can be divided into the following two categories (Huang et al., 2019, 2020). In the first category, some approaches based on data fusion strategy have been adopted, such as principal component analysis (PCA), multi-view embedding as well as multi-kernel learning (MKL) (Huang et al., 2019). More specifically, these approaches are applied to combining structural and functional network properties, which can reveal the balance of local and global efficiency between structural and functional networks (Rudie et al., 2013). Multi-kernel technology has been proved as an effective way in fusing multi-modal data (Zhang et al., 2011), and many experiments were done to demonstrate that the more discriminatory results can achieve than using only single modality (Zhang et al., 2011; Dyrba et al., 2015). In the second category, the commonly used strategy is using one of the modality to assist another modality (Huang et al., 2020). However, this strategy does not make full use of the complementary information between the two modalities. What is more, most of the existing research ignore the global brain region correlation and the information of nodes. Although some papers propose to consider node information, only some simple topology attribute measurement methods, such as clustering coefficients or average degree are utilized. More potential and significant node information is ignored, which can be extracted by higher-order methods. The difference between such two mechanisms is depicted in Figures 1A,B.

On the one hand, in the brain network construction, the previous fusion brain methods construct the functional and structural brain networks independently, which cannot comprehensively reflect the interaction between structural and functional image data. On the other hand, in the feature extraction of brain network, the previous methods mainly analyze the connectivity features or the simple topological features, such as clustering coefficients or average degree, from the graph of the brain network. Different from above two categories, we seek to develop a multi-modal jointly construction method shown in Figure 1C, in which the interaction between functional and structural data promotes the discovery of hybrid structural and functional connectivity and high-order node information of the network can be obtained.

In this paper, we proposed a unified multi-modal brain network (UM2BN) construction and fusion method. First, we make spatial calibration, and then build the correlation model among all brain regions with functional data by low-rank representation, so that the global brain region correlation can be captured. Simultaneously, the local manifold with structural data is embedded into this model, so the local structural information can be preserved by manifold learning. Second, the PageRank algorithm is adaptively used to evaluate the significance of different brain regions. Finally, a multi-kernel strategy is utilized to merge the connectivity and node significance information from the constructed unified network for classification.

The main contributions can be summarized as the following four folds:

1. A unified framework to construct brain network by combing both FC and SC is proposed, in which the space alignment and brain network construction are carried out under the same framework and promote each other.
2. The relationship between multiple brain regions can be comprehensively considered instead of only considering two brain regions by adding a low rank constraint. And the local structural information is preserved by manifold learning.
3. The significant node information is adaptively extracted by PageRank algorithm from the unified brain network. Compared with only using simple attributes such as clustering coefficient or average degree, significant high-order node information can be obtained, which may help to capture the slight change in brain network.
4. In order to solve the data heterogeneity problem, an effective multi-kernel technology is utilized to fuse information of connectivity as well as node importance for classification.

The experimental results show that, compared with a series of previous brain network analysis approaches, our approach can achieve a promising performance in the diagnosis of epilepsy on a real epilepsy dataset. A preliminary version of this work has been reported (Yang et al., 2020).

The rest of this paper is organized as follows. In section 2, we introduce related works. Then, we present the proposed multi-modal brain network jointly construction and fusion method in section 3. Section 4 introduces materials used in the study and provides the experimental results on epilepsy dataset. In section 5, we give an analysis of the experimental results. Finally, we summarize our work in section 6.

## 2. Related Works

### 2.1. Pearson Correlation Based Brain Network Construction

Functional magnetic resonance imaging (FMRI) uses blood oxygenation level dependent (BOLD) changes in brain blood flow and oxygen consumption to detect the activity of neurons. Because of its high temporal and spatial resolution, fMRI is widely used in the field of brain functional network research. Pearson-based method is the most widely used in functional network construction methods. Suppose there are two brain regions, whose BOLD signals are denoted by the vectors x and y. The connectivity strength between these two brain regions is measured by


(1) $$\begin{array}{l} \rho =\frac{Cov\left(\text{x},\text{y}\right)}{\sqrt{Var\left(\text{x}\right)Var\left(\text{y}\right)}} \end{array}$$

where *Cov*(**x, y**) denotes the covariance of **x** and **x**, and *Var*(**x**) and *Var*(**y**) denote the variance of **x** and the variance of **y**. For each pair of the regions, its connectivity strength can be calculated by above correlation. All the connectivities from the same subject form a brain network. As can be seen from the above description, the connectivity calculation in Pearson-based method only consider simple pairwise relationship of its attached brain regions, which may ignore the latent influence of other brain regions.

### 2.2. PageRank Algorithm

With the rapid development of the information technology, the number of users and web pages in the network grows very quickly. The network topology structure that reflects the relationship between users and web pages are becoming more and more complex, making it more difficult to provide users with high quality, relevant web pages based on user queries (Pandurangan et al., 2002). To address this issue, many algorithms have been proposed, among which PageRank algorithm is widely used because it can well reflect the high-order information of nodes (Pandurangan et al., 2002). The PageRank algorithm is based on two hypotheses. One is quantitative hypothesis: The more nodes connected to one node, the more important one node is. The other is quality hypothesis: If one node is more important, then other nodes connected to it are also more important. According to Gleich (2015), PageRank algorithm can be used not only in web learning, but also in social networks and bioinformatics. For example, Markovich et al. (2017) and Roul and Sahoo (2021) are both typical applications in web learning. Priyanta et al. (2019) has proved that PageRank algorithm can be used as an important tool for social network analysis. Jiang et al. (2017) use PageRank to diffuse information on two-layer graph model in protein structure analysis, and Liu et al. (2020) use PageRank to move the homologous proteins of query proteins to the neighbors of the query proteins in a protein similarity network. The PageRank algorithm indicates that if a node has important links to it, its links to other nodes are also significant (Xing and Ghorbani, 2004). Therefore, both the brain network and Web network can be abstracted as graph model, which is composed of a series of nodes and connectivities. The higher-quality pages point to page A, the more important page A is. In graph model, brain network and Web network have similar properties. Brain areas and brain connectivities correspond to pages and links in the Web network, respectively. The importance of a brain region in a brain network is related to the importance of other brain areas transferred by connectivities. If a brain region is connected to a more important brain region, its importance will be higher. Therefore, in our model, we use the PageRank algorithm to evaluate the similarity of brain regions. The advantage of this method is that the importance of global brain regions and brain connections are considered in the model calculation. It is suitable to apply PageRank algorithm in brain network analysis, because brain network also has these properties (Gleich, 2015).

## 3. Proposed Method

### 3.1. Notation

Denote boldface uppercase letter as a matrix (e.g., **X**), boldface lowercase letter as a vector (e.g., **x**), and lowercase letter as a scale (e.g., x). Further, we summarize the important symbols and definitions used in this article in Table 1.

**Table 1:** Notations and descriptions.

**Notation**	**Definition**
**X**	The feature matrix of fMRI time series, **X** = [**x**_1_, **x**_2_, …, **x**_*N*_].
**G**	The DTI matrix reflects the physical connections.
**D**	The degree matrix of **G**, ${\text{D}}_{ii}=\underset{i}{\sum }{\text{G}}_{ij}$ or $\underset{j}{\sum }{\text{G}}_{ij}.$
**L**	The Laplacian matrix of **G**, **L** = **D**−**G**.
**W**	The brain network matrix.
**U**	The projection matrix.
*tr*(**X**)	The trace of matrix **X**.
**X** ^*T*^	The transpose matrix of **X**.
**X**_*ij*_	The *i*th row and *j*th column element of matrix **X**.
**x** ^*i*^	The *i*th row of matrix **X**.
**x**_*j*_	The *j*th column of matrix **X**.
∥ · ∥_*_	The trace or nuclear norm of a matrix.
∥ · ∥_2_	The *l*_2_-norm.
∥ · ∥_*F*_	The Frobenius norm of the matrix.
〈**x, y**〉	The inner product between vector **x** and vector **y**.

### 3.2. Problem Formulation of Unified Brain Network

Our approach aims to learn a unified brain network representation combing structural connectivity and brain activities for disease diagnosis. More specifically, we utilize the matrix **W** ∈ ℝ^*N* × *N*^ to represent the unified brain network, the entry of **W** is **W**_*ij*_, which reflects not only the information of FC between *i*th brain region and *j*th brain region, but also the information from SC. Suppose ${\text{x}}_{i}\in {\mathbb{R}}^{K\times 1}$ is a feature vector of fMRI time series for *i*th brain region, **X** = [**x**_1_, **x**_2_, …, **x**_*N*_] is the feature matrix of brain connection, in which *N* indicates the number of brain regions and *K* indicates the number of time points of time series. The matrix **G** ∈ ℝ^*N*×*N*^ reflects the physical connections, whose entry **G**_*ij*_ ≥ 0 is the physical fiber quantity between brain region *i* and brain region *j*. The detail steps for constructing the unified brain network with functional and structural data proposed in this paper are as follows.

First, we suppose the feature matrix of brain activities can be linearly represented by the weight matrix **W**. It can be expressed by **X** = **XW**, and further written as


(2) $$\begin{array}{l} \underset{\text{W}}{\min}∥\text{X}-\text{X}\text{W}{∥}_{F}^{2} \end{array}$$

where ${\text{x}}_{i}\in {\mathbb{R}}^{K\times 1}$ is a feature vector of fMRI time series for *i*th brain region. In order to make (2) better depict the correlation between brain regions, space alignment method is adopted. Inspired by Zhang et al. (2016b), we introduce a projection matrix **U** ∈ ℝ^*M*×*K*^ to transform the original *k*-dimensional data into *m*-dimensional compact representations $\tilde{\text{X}}$. It is expressed by $\tilde{\text{X}}=\text{U}\text{X}$, where $\tilde{\text{X}}\in {\mathbb{R}}^{M\times N}$ and **X** ∈ ℝ^*K*×*N*^. Combined with (2), the model can be expressed as


(3) $$\begin{array}{l} \underset{\text{W}}{\min}∥\tilde{\text{X}}-\tilde{\text{X}}\text{W}{∥}_{F}^{2}+∥\text{U}{∥}_{F}^{2}\;s.t.\;\tilde{\text{X}}=\text{U}\text{X} \end{array}$$

where ∥ · ∥_*F*_ is utilized to constrain the projection matrix **U**.

Noteworthy, we make space alignment and brain network construction under a unified framework, so the two can promote each other for constructing a more precise brain network structure. Then, in order to consider the global brain region information, the low-rank (Han et al., 2019; Yu et al., 2019; Wang and Guo, 2020) constraint is introduced.

Rank of matrix **W** is a small number based on the assumption that each brain region can be approximately represented by a combination of only a few other brain regions. So objective function can be defined as


(4) $$\begin{array}{l} \underset{\text{W}}{\min}∥\tilde{\text{X}}-\tilde{\text{X}}\text{W}{∥}_{F}^{2}+rank\left(\text{W}\right)+∥\text{U}{∥}_{F}^{2}\;s.t.\;\tilde{\text{X}}=\text{U}\text{X} \end{array}$$

Because rank minimization problem is non-convex. Inspired by Wang et al. (2018, 2019), (4) is reformulated to


(5) $$\begin{array}{l} \underset{\text{W}}{\min}∥\tilde{\text{X}}-\tilde{\text{X}}\text{W}{∥}_{F}^{2}+∥\text{W}{∥}_{*}+∥\text{U}{∥}_{F}^{2}\;s.t.\;\tilde{\text{X}}=\text{U}\text{X} \end{array}$$

where ∥ · ∥_*_ is the trace or nuclear norm of a matrix. In addition, because DTI can reflects physical connectivity between functionally related gray matter regions, the matrix **G** is introduced to make the correlation model more discriminative. It has been proved that the foundation of FC is SC (Honey et al., 2009; Stam et al., 2016). Thus, we assume that the more fiber bundles exist between *i*th brain region and *j*th brain region, the closer the distance between **w**_*i*_ and **w**_*j*_ is. Inspired by He and Niyogi (2004), construct the following objective function:


(6) $$\begin{array}{l} \min\underset{i,j}{\sum }{\text{G}}_{ij}∥{\text{w}}_{i}-{\text{w}}_{j}{∥}^{2} \end{array}$$

On the whole, the final objective function is defined to jointly minimizing the above problem


(7) $$\begin{array}{l} \underset{\text{W}}{\min}∥\tilde{\text{X}}-\tilde{\text{X}}\text{W}{∥}_{F}^{2}+\alpha ∥\text{W}{∥}_{*}+\beta \underset{i,j}{\sum }{\text{G}}_{ij}∥{\text{w}}_{i}-{\text{w}}_{j}{∥}^{2}\\ +\gamma ∥\text{U}{∥}_{F}^{2}\\ s.t.\;\tilde{\text{X}}=\text{U}\text{X} \end{array}$$

where α, β, and γ are positive scalars weight the corresponding terms in (7). $\underset{i,j}{\sum }{\text{G}}_{ij}∥{\text{w}}_{i}-{\text{w}}_{j}{∥}^{2}$ can be rewritten as *tr*(**WLW**^*T*^), where **L** = **D**−**G** (Xu et al., 2015; Yu et al., 2019). Thus, we transform the objective function as


(8) $$\begin{array}{l} \underset{\text{W}}{\min}∥\tilde{\text{X}}-\tilde{\text{X}}\text{W}{∥}_{F}^{2}+\alpha ∥\text{W}{∥}_{*}+\beta tr\left(\text{W}\text{L}{\text{W}}^{T}\right)+\gamma ∥\text{U}{∥}_{F}^{2}\\ s.t.\;\tilde{\text{X}}=\text{U}\text{X},\text{L}=\text{D}-\text{G} \end{array}$$

**Figure 2:**
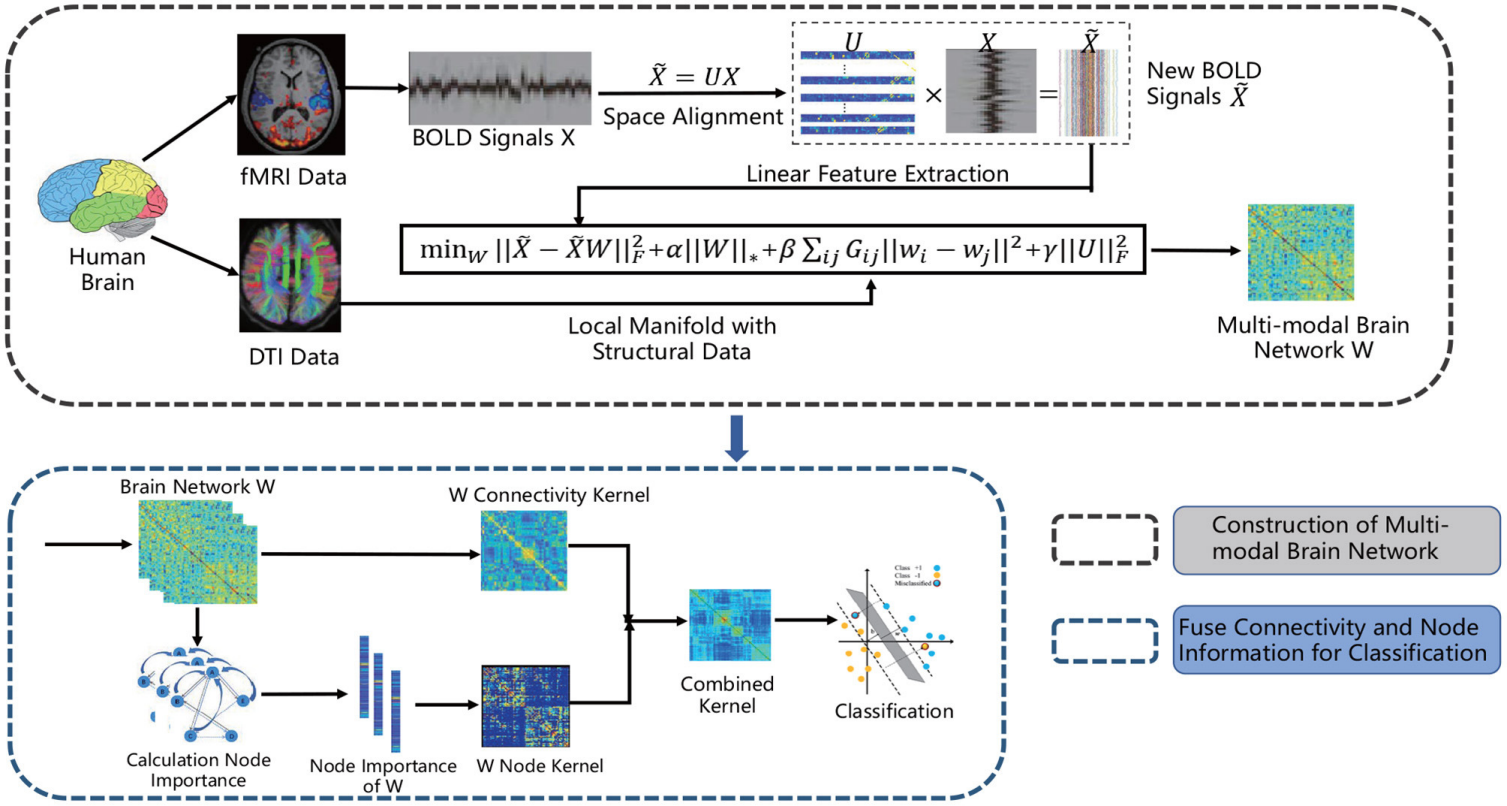
The flow chart of our proposed method for constructing unified brain network with functional and structural data and fusing connectivity as well as node information for classification.

The matrix **W** is the solution above problem, and the unified brain network represented by it contains the information of both FC and SC. Figure 2 gives a schematic illustration of our proposed method for constructing multi-modal unified brain network with significant information of nodes.

This paper is an extension of the work in MICCAI (Yang et al., 2020), but we need to point out that it has improvement in methodology and carried out more extensive experiments to evaluate the performance. The original method was performed in original feature space, and this work is conducted in feature space aligned by the projection shown in Equation (8). Although both of these two methods represent the signal from one brain region by signals from other brain regions, the choice of feature space directly affects the performance of the above representation model. The space alignment and brain network construction are carried out under the same framework and promote each other. In other words, the feature space of correction can help to establish more effective brain network, and the latter can also provide guidance for correction.

### 3.3. Alternating Optimization Algorithm

In order to solve this problem, some alternative optimization methods can be adopted. Here, the alternating direction method of multipliers (ADMM) algorithm (Xu et al., 2015) is utilized. First of all, we make the problem separable by introducing two auxiliary variables. And then (8) can be reformulated as


(9) $$\begin{array}{l} \underset{\text{W}}{\min}∥\tilde{\text{X}}-\tilde{\text{X}}\text{W}{∥}_{F}^{2}+\alpha ∥\text{P}{∥}_{*}+\beta tr\left(\text{Q}\text{L}{\text{Q}}^{T}\right)+\gamma ∥\text{U}{∥}_{F}^{2}\\ s.t.\;\tilde{\text{X}}=\text{U}\text{X},\text{L}=\text{D}-\text{G},\text{P}=\text{W},\text{Q}=\text{W} \end{array}$$

We solve problem (9) by minimizing the following augmented Lagrange multiplier (ALM) function *L*


(10) $$\begin{array}{l} L(W,P,Q,U,\tilde{X},Y_{1},Y_{2},Y_{3})=∥\tilde{X}-\tilde{X}W{∥}_{F}^{2}+\alpha ∥P{∥}_{*}+\beta tr(Q\\ LQ^{T})+\gamma ∥U{∥}_{F}^{2}+\langle Y_{1},P-W\rangle +\langle Y_{2},Q-W\rangle +\langle Y_{3},\tilde{X}-UX\rangle \\ \;+\frac{\mu }{2}(∥P-W{∥}_{F}^{2}+∥Q-W{∥}_{F}^{2}+∥\tilde{X}-UX{∥}_{F}^{2}) \end{array}$$

where **Y**_1_, **Y**_2_, and **Y**_3_ are Lagrange multipliers, and μ > 0 is a penalty parameter. ADMM algorithm is an iterative method that solves for each variable in a coordinate descent manner. The update formulas for those variables are as follows:


(11) $$\begin{array}{l} {\text{W}}^{*}={\left(\text{K}+{\text{K}}^{T}+2\mu \text{I}\right)}^{-1}\left(\left(\text{K}+{\text{K}}^{T}\right)+\mu \left(\text{P}+\text{Q}\right)+\left({\text{Y}}_{1}+{\text{Y}}_{2}\right)\right) \end{array}$$

(12) $$\begin{array}{l} {\text{P}}^{*}=\vartheta_{\alpha /\mu }\left(\text{W}-\frac{{\text{Y}}_{1}}{\mu }\right) \end{array}$$

(13) $$\begin{array}{l} {\text{Q}}^{*}=\left(\mu \text{W}-{\text{Y}}_{2}\right){\left(\beta \left(\text{L}+{\text{L}}^{T}\right)+\mu \text{I}\right)}^{-1} \end{array}$$

(14) $$\begin{array}{l} {\text{U}}^{*}=\left(\mu \tilde{\text{X}}{\text{X}}^{T}+{\text{Y}}_{3}{\text{X}}^{T}\right){\left(2\gamma \text{I}+\mu \text{X}{\text{X}}^{T}\right)}^{-1} \end{array}$$

(15) $$\begin{array}{l} {\tilde{\text{X}}}^{*}=\left(\mu \text{U}\text{X}-{\text{Y}}_{3}\right){\left(2\text{I}-2\text{W}-2{\text{W}}^{T}+2\text{W}{\text{W}}^{T}+\mu \text{I}\right)}^{-1} \end{array}$$

where $\vartheta_{\lambda }\left(\text{X}\right)=\text{U}{\text{S}}_{\lambda }\left(\Sigma \right){\text{V}}^{T}$ is a thresholding operator with respect to a singular value λ; **S**_λ_(Σ_*ij*_) = *sign*(Σ_*ij*_)max(0, |Σ_*ij*_ − λ|) is the soft-thresholding operator; **X** = **U**Σ**V**^*T*^ is the singular value decomposition of **X**.

Multipliers **Y**_1_,**Y**_2_,**Y**_3_ and iteration step-size ρ(ρ > 1) are updated by using (16),


(16) $$\{\begin{array}{c} Y_{1}=Y_{1}+\mu (P-W)\\ Y_{2}=Y_{2}+\mu (Q-W)\\ Y_{3}=Y_{3}+\mu (\tilde{X}-UX)\\ \mu =\min(\rho \mu ,\mu_{max}) \end{array}$$

We summarize the process of solving (9) in Algorithm 1.

**Algorithm 1:** Solving Problem (8) by ADMM

**Input: X,G**, m, α, β, andγ;
**Initialization:** $Y_{1}=Y_{2}=Y_{3}=0;\mu =0.1;\mu_{max}=10^{-7};\;$
ρ = 1.01;ϵ = 10^−7^;
**While** not converged **do**
1. Fix the other variables and update **W** by using (10).
2. Fix the other variables and update **P** by using (11).
3. Fix the other variables and update **Q** by using (12).
4. Fix the other variables and update **U** by using (13).
5. Fix the other variables and update $\tilde{X}$ by using (14).
6. Update the multipliers and parameters by (15).
7. Check the convergence conditions
$∥P-W{∥}_{\infty }<\epsilon ;∥Q-W{∥}_{\infty }<\epsilon ;∥\tilde{X}-UX{∥}_{\infty }<\epsilon$.
**End While**
**Output: *W***

### 3.4. Node Information

**Figure 3:**
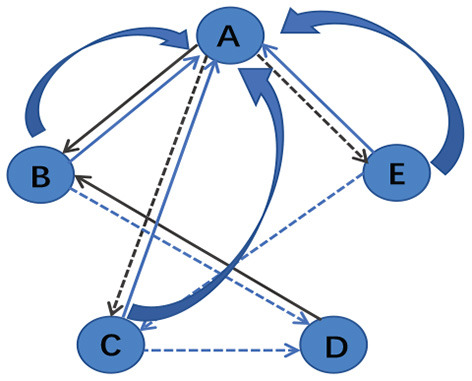
An example diagram showing the calculation principle of PageRank algorithm.

After building the unified brain network, PageRank algorithm is utilized to evaluate the importance of different brain regions. According to Yan and Ding (2011), a simplified version of PageRank is defined as $s_{u}=\underset{v\in P\left(u\right)}{\sum }\frac{s_{v}}{N_{v}}$, where *u* indicates a node, and *P*(*u*) is the set of nodes that connect to node *u*. *N*_*v*_ denotes the number of links of node *v* and *s*_*u*_ represents the score of node *u*. The calculation process of the algorithm is shown in Figure 3. For example, in Figure 3, *P*(*A*) = {*B, C, E*} and *P*(*B*) = {*A, D*}, and thus *s*_*A*_ = *s*_*B*_/2+*s*_*C*_/2+*s*_*E*_/2 and *s*_*B*_ = *s*_*A*_/3+*s*_*D*_.

Suppose the vector $\text{s}=\left[s_{1},s_{2},...,s_{n}\right]\in {\mathbb{R}}^{N\times 1}$ reflects the importance of different brain regions. Its entry *s*_*u*_ is the score of the *u*th brain region with restriction $\overset{N}{\underset{u=1}{\sum }}s_{u}=1$. And the larger the number of *s*_*u*_ is, the more important the *u*th brain region is. First of all, we transform the matrix **W** into a matrix **B** ∈ {0, 1}^*N*×*N*^ through a threshold factor *r* (if **W**_*vu*_ ≥ *r*, then the corresponding entry **B**_*vu*_ is set to one; otherwise, the entry **B**_*vu*_ is set to zero). And the value of vector **s** is initialized as $\text{s}=\left[\frac{1}{N},\frac{1}{N},...,\frac{1}{N}\right]$.


(17) $$T_{vu}=\{\begin{array}{cc} \frac{1}{\sum_{i=1}^{N}B_{ui}}, & if\;B_{uv}=1\\ 0, & if\;B_{uv}=0 \end{array}$$

Then, we introduce a transfer matrix **T** ∈ ℝ^*N*×*N*^ to iterative update vector **s**, based on the iterative updating formula for **s** = **T** × **s**, until the convergence condition is satisfied.

### 3.5. Multi-Kernel SVM Classification

After obtaining the connectivity and node importance information from the constructed unified network, a multi-kernel strategy is adopted to fuse these heterogeneous information. The reasons why we adopt the multi-kernel fusion method are as follows. The previous study (Zhang et al., 2011; Huang et al., 2019) has shown that multi-kernel can effectively fuse multi-modal data and has been successfully applied into the diagnosis of brain diseases. For example, Zhang et al. (2011) used multi-kernel to fuse the multi-modal brain image data for Alzheimer's disease classification. Huang et al. (2019) employed multi-kernel to combine functional and structural connectivity features, and applied it into epilepsy classification. In addition, the multi-kernel method can handle heterogeneous multimodal data fusion by kernel trick. In our method, the obtained brain region features and brain connectivities are with different dimensionalities. Considering above two aspects, we adopted the multi-kernel method to fuse the multi-modal feature in brain network. The first modality is the connectivity of unified brain network, and the second modality is the node importance information of unified brain network. More specifically, $k\left({\text{x}}_{i},{\text{x}}_{j}\right)=\underset{m}{\sum }c_{m}k^{\left(m\right)}\left({\text{x}}_{i}^{\left(m\right)},{\text{x}}_{j}^{\left(m\right)}\right)$ is defined as a mixed kernel between the multi-modal training sample **x**_*i*_ and **x**_*j*_, and $k\left({\text{x}}_{i},\text{x}\right)=\underset{m}{\sum }c_{m}k^{\left(m\right)}$$\left(x_{i}^{\left(m\right)},x^{\left(m\right)}\right)$ is defined as the mixed kernel between the multi-modal training sample **x**_*i*_ and the test sample **x**. $\underset{m}{\sum }c_{m}=1$ is restricted and a coarse-grid search is adopted to search the optimal values. Then, we fuse two kernels into a mixed kernel and a standard SVM classification algorithm is performed.

## 4. Experiments

### 4.1. Materials and Preprocessing

Before experiments, we collected raw rs-fMRI data and DTI data from 306 peoples. Including 114 normal controls (NC), 103 patients with frontal lobe epilepsy (FLE), and 89 patients with temporal lobe epilepsy (TLE). All the subjects are with right-handed.The information of the dataset are shown in Table 2. Those data are collected by Siemens Trio 3T scanner at Jinling Hospital, Nanjing, China. The scan parameters are as follows: repetition time = 2,000*ms*; echo time = 30*ms*; and flip angle =90°; 30 transverse slices; field of view (FOV)= 240 × 240*mm*; slice thickness = 4*mm*; interstice gap = 0.4*mm*; voxel size = 3.75 × 3.75 × 3.75*mm*; DTI scans were obtained by using spin echo-based echo planar imaging sequence. The scan parameters are as follows: repetition time = 6,100*ms*; echo time = 93*ms*; flip angle = 90°; field of view = 240 × 240*mm*; matrix size = 256 × 256; voxel size = 0.94 × 0.94 × 3*mm*; 45 slices.

**Table 2:** The detail information of the epilepsy dataset.

**Data**	**Quantity**	**M/F^a^**	**Age range**	**Mean age**
NC	114	58/56	20–38	26.2
FLE	103	53/50	17–51	24.1
TLE	89	44/45	17–51	25.9

a *M/F indicates Male/Female*.

The functional network is constructed through fMRI and the structural network through DTI. The SPM8 in the DPARSF toolbox is utilized to pre-process all rs-fMRI images. Specifically, slice time are collected, corrected, rearranged, and normalized to the EPI template to obtain the initial functional time series. Then, the de-trending process is performed to remove spurious sources of variance. Utilizing the AAL atlas, we divided the resulting volumes consist of 240 time points into 90 regions of brain interest (ROIs), so that those time series reflect the information about brain activities. The DTI data are processed by the PANDA suite. First, the FSL toolbox is utilized to correct the DTI distortion, remove the eddies, and extract the brain mask from the non-diffusion weighted (B0) image. Then, the TrackVis is used to obtain fiber images by deterministic tracking method, and defined anatomic areas using AAL conventions based on each subject's co-registered T1 images. Finally, the quantity of fibers can naturally be viewed as the edge strength of the structural network.

### 4.2. Competing Methods

In order to verify the effectiveness of our proposed method, we compare it with several classical and state-of-the-art methods. These methods fall into three categories: fMRI-based methods, DTI-based methods, fMRI- and DTI-based methods. More specifically, fMRI-based methods are Pearson coefficient (PC) (Betzel et al., 2016), low-rank sparse representation (LSR) (Qiao et al., 2016), weighted sparse group representation (WSGR) (Yu et al., 2017), Strength and Similarity GSR (SSGSR) (Zhang et al., 2019), high-order FC (HOFC) (Chen et al., 2016), topographic FC (tHOFC) (Zhang et al., 2016a), Graph-CNN (GCNN) (Mao et al., 2018), and Siamese-GCN (SGCN) (Ktena et al., 2017). DTI-based methods are Graph kernel (GK) (Kang et al., 2012), Graph-CNN (GCNN) (Mao et al., 2018). fMRI- and DTI-based methods are multi-kernel (MK) (Dyrba et al., 2015), our methods without space alignment, our method without node importance information, and our proposed methods (JCFBN). In addition, canonical analysis (CCA), kernel-canonical analysis (KCCA), and manifold regularized (M2TFS) algorithms are all utilized to merge multi-modal data. We briefly summarize these comparison methods as follows.

In the PC (Betzel et al., 2016) method, the functional connectivity matrix is defined by Pearson's correlation coefficient. Then, we extract the upper triangular element of the functional connectivity matrix and compress it into a vector for each subject. Finally, the standard SVM is exploited for classification.

In the LSR (Qiao et al., 2016) method, a functional brain network (FBN) is constructed which jointly learns from partial correlation and sparse representation. Then, based on the matrix-regularized network learning framework, we further formulate it as a sparse low-rank graph learning problem. Finally, *t*-test is used for feature selection and SVM is adopted for classification.

The WSGR (Yu et al., 2017) method ensures the construction of more biologically meaningful brain network by integrating connectivity strength, group structure, and sparsity. In contrast to traditional sparse representation, a connectivity strength weight matrix is defined based on Pearson's correlation matrix for *l*-1 norm, and a group partition for *l*-21 norm is added in constructing brain networks. Then, a linear SVM is exploited for classification.

The SSGSR (strength and similarity guided GSR) (Zhang et al., 2019) method, which exploits both BOLD signal temporal correlation-based “low-order” FC and intersubject LOFC-profile similarity-based “high-order” FC as two priors to jointly guide the GSR-based network modeling. Then, the upper triangular element of the GSR-based network is extracted, and compressed into a vector. Finally, a linear SVM is used for classification.

In HOFC (Chen et al., 2016) method, an FC profile is calculated for each brain region first. Then, based on these FC profiles, a second layer of correlations is computed between all pairs of brain regions to generate an HOFC network. Then, for each subject, the upper triangular element of the HOFC network is extracted, and compressed it into a vector. Finally, a standard SVM is used.

The tHOFC (Zhang et al., 2016a) method is similar to HOFC method. More specifically, topographical profile similarity-based HOFC (tHOFC) is one types of HOFC method. Both of them have the idea of computing “correlation of correlations.” Nonetheless, instead of measuring the similarity of the original rs-fMRI signals with the traditional FC, tHOFC measures the similarity of LOFC profiles between each pair of brain regions.

In the GCNN (Mao et al., 2018) model, a specific convolutional operator is designed for brain network which applies a row scanning on adjacent matrix to generate the feature map. Classification results are acquired by the softmax function based on these feature maps. This GCNN model can deal with both FC and SC. It is worth noting that the FC is defined by Pearson's correlation coefficients, while the SC is obtained by using the same approach as our proposed method.

In the SGCN (Ktena et al., 2017) method, a pair of brain networks are defined by FC matrices and a common graph structure is defined by the anatomy of brain. Then, the common graph structure is used for spectral graph convolutional networks. The model including an inner product layer, which is used to combined node representations from two brain networks, and a single fully connected output layer is used to output the similarity between brain networks, subsequently. Finally, the KNN classifier is exploited to disease diagnosis.

In the GK (Kang et al., 2012) method, a random walk graph kernel is used to measure the similarity between brain networks based on the number of common walks in the two networks. Then, a kernel matrix is constructed by these pairwise similarity. At last, the kernel matrix is fed into the SVM for disease diagnosis directly.

In the MK (Dyrba et al., 2015) method, a linear kernel matrix is calculated based on the feature vectors used in the PC methods, while the graph kernel matrix is calculated same as the GK method. Then, the two kernel matrices are linearly combined into a mixed kernel matrix by grid search to find the optimal parameters. At last, the standard SVM algorithm is performed for classification.

The CCA algorithm is used to fuse connectivity and node importance information of the brain network. These multi-modal data of different dimensions are mapped to the same subspace, giving them the same dimension and the greatest linear correlation. Finally, a more discriminative set of features is obtained from multi-modal information.

The KCCA algorithm is an extension of CCA algorithm, and is also used to fuse multi-modal data, namely the connectivity of the brain network and the node information. Unlike the CCA method, it uses a kernel function to map the raw data into a higher-dimensional space and then looks for nonlinear relationships between different modalities.

In the M2TFS method, the functional connectivity matrix defining by Pearson correlation coefficient is viewed as the first modality, the DTI data are viewed as the second modality. First, M2TFS denotes the feature learning on each modality as a single task. Then, it uses group-sparsity regularizer to capture the intrinsic relatedness among multiple tasks. Furthermore, a new manifold-based Laplacian regularizer is introduced to preserve the data distribution information from each task. Finally, a multi-kernel SVM method to fuse multi-modal data for classification.

To verify the effect of the space alignment as well as node importance information, we omit these two parts respectively, and then calculate the classification accuracies. In the first case (i.e., our method without space alignment), we directly use the original fMRI data to construct the unified brain network. In the second case (i.e., our method without node importance), we directly utilize the information of brain network connectivity for disease diagnosis without taking into account the node information.

### 4.3. Experimental Setup

In order to evaluate the performance of our proposed method, we apply it to four different classification experiments, including NC vs. FLE, NC vs. TLE, FLE vs. TLE, and NC vs. (FLE and TLE). We use the following measures to ensure the fairness of the comparison. For parameter setting, we perform our method and the comparison methods with grid search parameter selection, and choose the one that performed best as the parameter. For the splitting of the dataset in experiment, we used five-fold cross-validation to verify the model's performance. The whole dataset was divided into five exclusive subsets, which have the same or similar size. The experiment was repeated for 5 times, the reported result was the average accuracy. Noting that it is necessary to ensure that the test set data is not used in the model training process. Classification accuracy (ACC) is used as an indicator to evaluate the classification performance.

### 4.4. Results on Epilepsy Data

Experimental results of all methods are summarized in Table 3. As can be seen from Table 3, in the four classification tasks, the accuracies of the proposed method are 73.3, 75.5, 67.9, and 75.0%, respectively. Compared with other methods, the method in this paper achieves the highest accuracies in the three tasks: NC vs. FLE, NC vs. TLE, NC vs. (FLE and TLE). Although SGCN algorithm has the highest classification accuracy in the FLE vs. TLE task, the discrepancy between it and the accuracy of our method 67.9% is relatively small. In addition, observing the Table 3, we can also draw the following three conclusions.

**Table 3:** Performance of proposed method and comparative methods.

	**Methods**	**NC:FLE**	**NC:TLE**	**FLE:TLE**	**NC:(FLE & TLE)**
	PC (Betzel et al., 2016)	62.1	63.9	51.7	65.6
	LSR (Qiao et al., 2016)	62.0	65.5	59.0	62.7
	WSGR (Yu et al., 2017)	61.7	70.9	44.9	63.8
fMRI based	SSGSR (Zhang et al., 2019)	62.6	67.0	56.8	64.9
methods	HOFC (Chen et al., 2016)	61.5	63.0	57.0	65.2
	tHOFC (Zhang et al., 2016a)	62.3	65.9	56.1	65.7
	GCNN (Mao et al., 2018)	55.8	67.5	61.4	67.0
	SGCN (Ktena et al., 2017)	67.3	74.5	**70.5**	69.3
DTI based	GK (Kang et al., 2012)	56.3	53.0	54.1	61.3
methods	GCNN (Mao et al., 2018)	51.6	54.5	56.4	62.6
	MK (Dyrba et al., 2015)	61.8	70.2	59.9	68.1
	CCA	62.6	67.3	59.2	68.6
	KCCA	64.6	68.5	60.7	66.7
fMRI & DTI	M2TFS	60.8	64.5	60.2	66.5
based methods	UBNfs (Yang et al., 2020)	71.3	75.1	69.0	71.9
	Our method I^a^	71.6	74.2	67.0	73.6
	Our method II^b^	69.0	72.0	63.8	71.5
	Our method	**73.3**	**75.5**	67.9	**75.0**

a *Our method I indicates that our proposed method without carrying space alignment and brain network construction under a unified framework*.

b *Our method II indicates that our proposed method without combining node importance information for classification*.

*The best result is highlighted in bold*.

First, the fMRI-based method achieved better results than the method based on only DTI data, indicating that fMRI data contained more effective information than DTI data in the epilepsy disease diagnosis tasks. In general, the multi-modal method combining fMRI and DTI data achieved higher classification accuracy than using only single modality. This indicates that the multi-modal classification method is indeed an effective method in brain disease diagnosis.

Second, the classification accuracies can be improved by combining the node information measured by PageRank algorithm. More specifically, we utilize our proposed method without node importance to classification, and the accuracies are only 69.0, 72.0, 63.8, and 71.5%. Compared with adding node importance information, the accuracies are relatively poor. This can indicate that the node importance of brain network measured by PageRank algorithm does contain important information, which is helpful for the diagnosis of epilepsy.

Third, the space alignment operation improves the final classification accuracies. Our goal is to construct a more precise brain network structure by making space alignment and brain network construction under a unified framework. The results show that 1.7, 1.3, 0.9, and 1.4% can be improved in the four classification tasks of NC vs. FLE, NC vs. TLE, FLE vs. TLE, NC vs. (FLE and TLE), respectively.

### 4.5. Comparison of Other Topological Attributes

**Figure 4:**
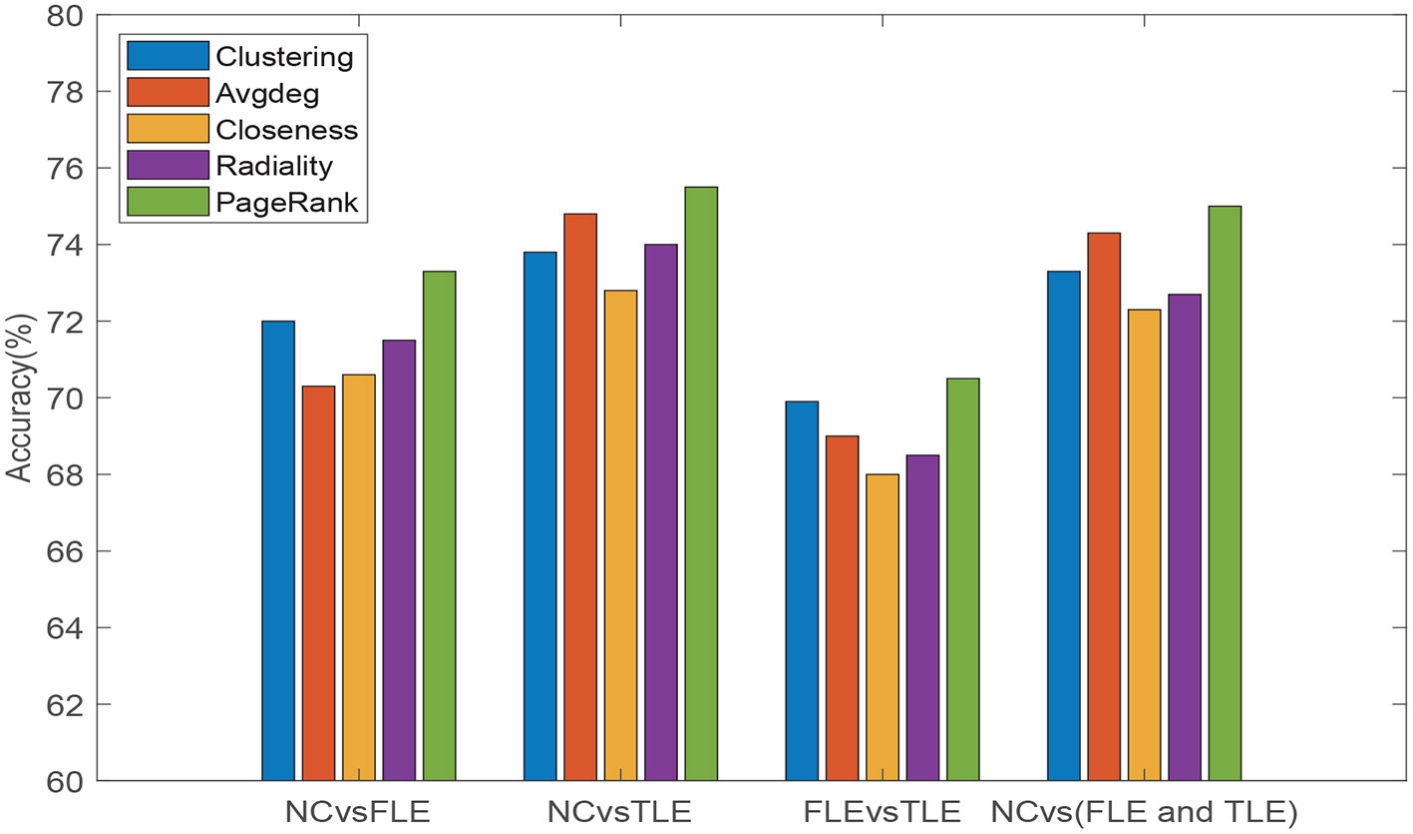
Classification accuracies of five different topological attribute measurement methods (Clustering, Avgdeg, Closeness, Radiality, and PageRank algorithm) on four different classification tasks (NC vs. FLE, NC vs. TLE, FLE vs. TLE, NC vs. (FLE and TLE)).

We also conducted experiment to verify that the PageRank algorithm introduced to evaluate the importance of different brain regions is reasonable and can achieve good results. Other commonly used topological attribute measures, e.g., Clustering coefficient (Clustering), Average degree (Avgdeg), Closeness centrality (Closeness), and Radiality are, respectively, used to evaluate the importance of brain regions, and the results of PageRank algorithm are compared. Specifically, we maintain the same experimental setup, changing only the method used to estimate the node information. The experimental results are reflected in Figure 4. As can be seen from Figure 4, we can see that, compared with other measurement methods, introduced PageRank algorithm achieves the highest accuracies in all four different experiments. Thus, it can be verified that the PageRank algorithm is reasonable and effective in the assessment of unified brain network node importance information.

### 4.6. Discussion on the Effect of Space Alignment and DTI Constraint

When the characteristic dimension is large, the distance between samples tends to be consistent, and the information of the relationship between samples is easy to be concealed. So it is usually necessary to reduce the dimension of the data in advance. However, this process is independent of the subsequent structural information mining and cannot guarantee the original data structure information contained in the feature space after dimension reduction. In order to solve this problem, we introduce space alignment into the construction process of unified brain network. Space alignment and brain network construction are carried out under a unified framework, and the two promote each other. On the one hand, a better feature space is beneficial to the mining of precise brain network structure information. On the other hand, precise brain structure information is also helpful to the selection of a better feature space. In addition, we use DTI to constrain the correlation between the two brain regions [expressed in (5)]. According to Honey et al. (2009) and Stam et al. (2016), a reasonable hypothesis is that the more fibers between brain region *i* and brain region *j*, the closer *W*_*i*_ and *W*_*j*_ is. Therefore, Equation (5) is constructed inspired by He and Niyogi (2004). In order to validate the rationality and effectiveness of space alignment and DTI constraint, we delete the linear feature extraction part and the DTI constraint part in the final objective function, respectively, but guarantee other experimental setup are the same. We show the classification accuracies in Table 4. As can be seen from Table 4, during the course of brain network construction, deleting any part of two in the objective function will reduce the classification accuracies. It shows that both space alignment and DTI constraint contribute to the more precise brain network structure. Results demonstrate that the two promote each other, which can improve the performance of disease diagnosis.

**Table 4:** Performance comparison of the proposed method and its variants.

**Methods**	**NC vs. FLE**	**NC vs. TLE**	**FLE vs. TLE**	**NC vs. (FLE and TLE)**
UM2BN-1*a*	71.6	74.2	67.0	73.6
UM2BN-2*b*	70.9	73.0	65.8	71.6
UM2BN-3*c*	70.4	72.5	65.0	70.5
UM2BN	**73.3**	**75.5**	**67.9**	**75.0**

a *UM2BN-1 indicates our method without carrying space alignment and brain network construction under a unified framework*.

b *UM2BN-2 indicates our method without DTI constraint*.

c *UM2BN-3 indicates our method with neither space alignment and DTI constraint*.

*The best result is highlighted in bold*.

## 5. Discussion

### 5.1. Analysis of Convergence

**Figure 5:**
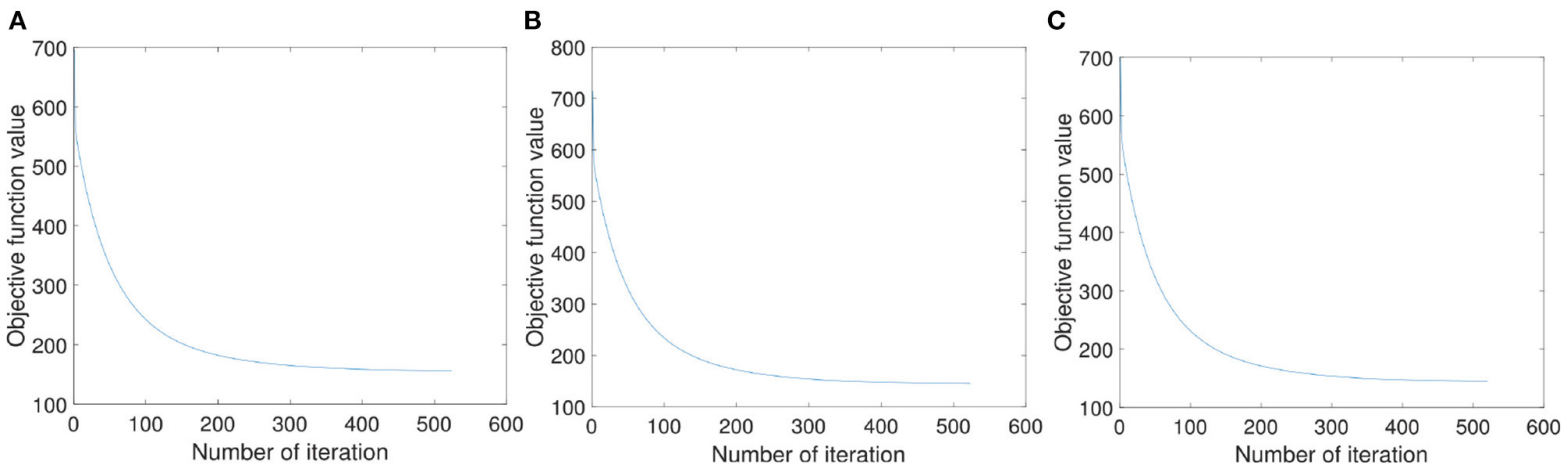
The convergence property of our proposed objection function. **(A–C)** Are the convergence curves with three random samples, respectively.

In order to verify the rationality of the solving process of the objective function (8), we randomly select three samples and, respectively, draw the convergence curve in Figures 5A–C. As can be seen from Figure 5 that with the increase of iteration times, the value of the objective function first decreases then tends to be stable. And it can be seen that our proposed method can converge to a certain value within 600 iterations.

### 5.2. Analysis of Parameter Sensitivity

**Figure 6:**
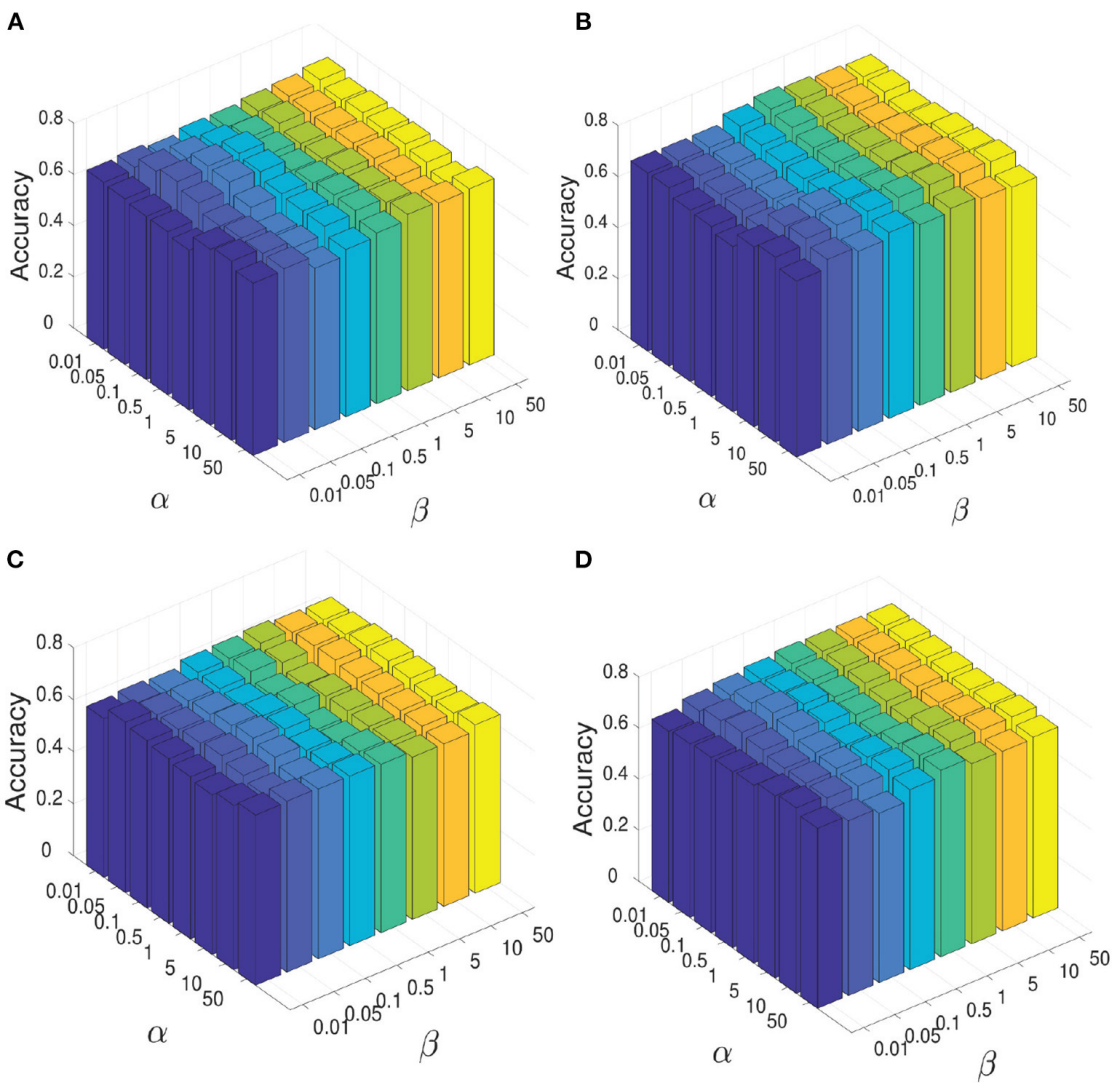
When constructing brain network, the influence of parameter α and parameter β on the all four classification tasks: NC vs. FLE, NC vs. TLE, FLE vs. TLE, NC vs. (FLE and TLE). **(A)** Shows the resutls on the task of NC vs. FLE. **(B)** Shows the resutls on the task of NC vs. TLE. **(C)** Shows the resutls on the task of FLE vs. TLE. **(D)** Shows the resutls on the task of NC vs. (FLE and TLE).

**Figure 7:**
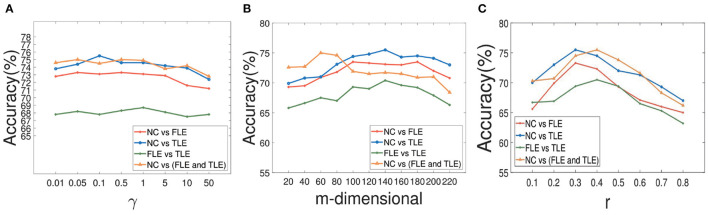
The results of our method with different parameters. The effect of parameter γ on our method with different classification tasks is reflected in **(A)**. And the impact of parameter *m* during the course of space alignment with different classification tasks is showed in **(B)**. The accuracies of our method with different threshold *r* on different classification tasks are displayed in **(C)**.

During the experiment, there are three hyperparameters that affect the construction of brain network, i.e., α, β, and γ. In order to study the influence of these parameters, we conduct the following experiments: First, we fix the parameter γ as the default value 1.0, study the influence of two hyperparameters α and β, and reflect the experimental results in Figure 6. Among them, both of α and β range from {0.01,0.05,…,50}. It can be seen that when the range of parameters α and β is within {0.01,0.05,…,50}, our proposed algorithm is relatively stable. After that, we set both parameters α and β as 1.0, study the influence of parameter γ on classification accuracies, and reflected the results in Figure 7A. It can be seen from Figure 7A that when the value range of γ parameter is {0.01,0.05,…,50}, the classification accuracies change only slightly for four different classification tasks. It can be concluded that the proposed method is relatively stable under different values of α, β, and γ. In addition, the parameter *m* used in the linear feature extraction will also affect the final experimental results. We also conduct experiments to study the influence of this parameter on the classification accuracies, as shown in Figure 7B. It can be seen that when parameter *m* changes within the range of {20, …, 220}, the classification accuracies of the four different tasks will also change. For the NC vs. FLE task, the optimal *m* value range is 100–180. For the NC vs. TLE task, the optimal *m* value range is 120–160. For the FLE vs. TLE task, the optimal *m* value is 100–160. And for the NC vs (FLE and TLE) task, the optimal *m* value is in the range of 60–80. Before using the PageRank algorithm to evaluate the importance of nodes in the brain network matrix W, a thresholding operation needs to be performed on the initial brain network. The parameter r is to control the sparsity of the network in thresholding. The larger the value of r is, the sparser the network after thresholding is. In the experiment, we control the value range of r in the range of [0.1, 0.2,…, 0.8], and determine the optimal value of r by searching the candidate set. The experimental results are shown in Figure 7C. As can be seen from this figure, the experimental accuracy is sensitive to the value of r. If the value of r is too large or too small, it will cause poor accuracies. The reason might be as follows. When the value of r is too large, there are too few or even no edges are preserved in the network, and the PageRank algorithm cannot extract the relationship among most of the nodes. When the value of r is too small, the network is very dense, which leads to average the importance of the node. Experimental results show that in the NC vs. FLE and NC vs. TLE tasks, the best value of r is 0.3; in the FLE vs. TLE and NC vs. (FLE and TLE) tasks, the best value of r is 0.4.

### 5.3. Analysis of Information Brain Regions

**Figure 8:**
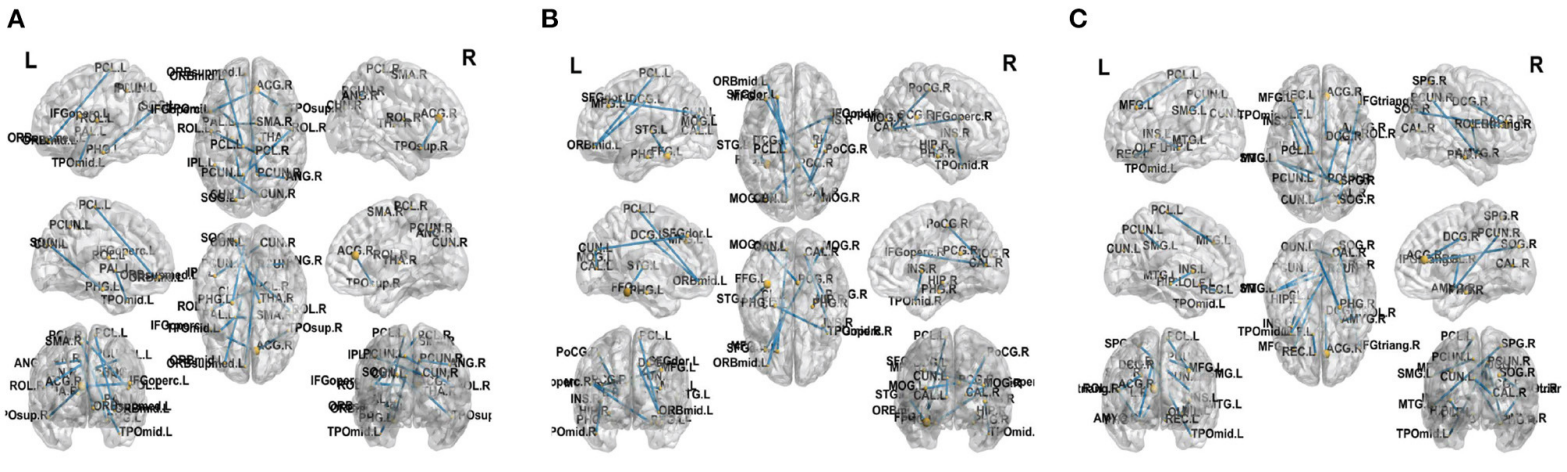
Top 12 significant alterations of connectivity between normal controls and patients with FLE **(A)**, normal controls and patients with TLE **(B)**, patients with TLE and patients with FLE **(C)**.

This work adopted the indicator proposed in Zhu et al. (2018) to evaluate the brain region importance. We establish the discriminant model from brain connectivities to labels by non-negative elastic-net sparse constraint. Then the connectivities can be ranked according to the value of the representation coefficients.In order to prove the effectiveness of the proposed method, we select top 12 significant alterations of connectivity and reported in Figure 8 for three classification tasks, respectively. It can be found that some brain connectivities involving brain regions such as Parahippocampal gyrus, Precuneus, and Superior temporal gyrus have significant changes. It means that these connectivities are the key to distinguish between normal people and FLE patients. There are similar findings in the literatures (Woodward et al., 2014; Zhang et al., 2019). Similarly, in the NC vs. TLE task, brain regions such as Parahippocampal and Amygdala are selected, which was supported by the work of Reinsberger et al. (2010) and Qiao et al. (2016). In the FLE vs. TLE task, some connectivities about brain regions such as Middle frontal gyrus, ParaHippocampal, and Amygdala have significant changes. Reinsberger et al. (2010) and Exner et al. (2002) have also mentioned this finding. Extensive evidence proved the effectiveness of the proposed method.

### 5.4. Limitations and Future Work

Although the experimental results show that our method has achieved good results in epilepsy diagnosis. But it still has some limitations. First, the nodes used in this study are defined by the AAL template, which divides the brain into only 90 ROIs. In the future, we will try to use other templates to divide the brain more finely. Second, PageRank algorithm is utilized to extract node information of the unified brain network, and then fuse connectivity and node information for classification. The experimental results show that, compared with other common topological attribute measurement methods, the PageRank algorithm can get better results. However, there are currently some variants of PageRank algorithm, such as weighted PageRank (Xing and Ghorbani, 2004). In future work, we will try to use variants of PageRank methods, which may improve classification accuracies. Third, in order to verify the effectiveness of our method, we apply it to the multi-modal epilepsy dataset. The experimental results show that our method achieves good results in four different classification tasks. In the future, we will consider extending our method to other brain disease diagnosis tasks to further explore the application value of our proposed method.

## 6. Conclusion

In this paper, a unified brain network construction algorithm is proposed, which is jointly learned from both functional and structural data, and make full use of complementary information between each other. In our method, we make the space alignment and multi-modal brain network construction under a unified framework, so that the two can promote each other. Instead of only considering two brain regions, we comprehensively consider the global brain regions relationship by low-rank constraint. And the local structural information can be preserved by extending the local manifold learning into this model. What is more, we take into account not only the connectivity, but also the node importance information of the unified brain network, extracted by PageRank algorithm. Finally, a multi-kernel strategy is utilized to solve the data heterogeneity problem and merge the connectivity as well as node information for classification. At last, we apply the proposed method (JCFBN) to the epilepsy diagnosis, and the experimental results show that our method can achieve a promising performance on all four classification experiments.

## Data Availability Statement

The raw data supporting the conclusions of this article will be made available by the authors, without undue reservation.

## Author Contributions

QZ and JY conceived the experiment and completed the manuscript. BX and LS preprocessed the data. ZH provided clinical guidance on biomarkers. DZ was responsible for data analysis. All the authors listed have made practical contributions to this work and agree with the manuscript.

## Funding

This work was supported in part by National Natural Science Foundation of China (Nos. 62076129, 61501230, 61732006, 61876082, and 61861130366), National Science and Technology Major Project (No. 2018ZX10201002), and the National Key R&D Program of China (Grant Nos. 2018YFC2001600 and 2018YFC2001602).

## Conflict of Interest

The authors declare that the research was conducted in the absence of any commercial or financial relationships that could be construed as a potential conflict of interest.

## Publisher's Note

All claims expressed in this article are solely those of the authors and do not necessarily represent those of their affiliated organizations, or those of the publisher, the editors and the reviewers. Any product that may be evaluated in this article, or claim that may be made by its manufacturer, is not guaranteed or endorsed by the publisher.
